# A microsatellite diversity analysis and the development of core-set germplasm in a large hulless barley (*Hordeum vulgare* L.) collection

**DOI:** 10.1186/s12863-017-0563-x

**Published:** 2017-12-06

**Authors:** Qijun Xu, Xingquan Zeng, Bin Lin, Zeqing Li, Hongjun Yuan, Yulin Wang, Zhasang, Nyima Tashi

**Affiliations:** 1 Institute of Agricultural Research, Tibet Academy of Agricultural and Animal Husbandry Sciences, Lhasa, 850002 China; 2 State Key Laboratory of Barley and Yak Germplasm Resources and Genetic Improvement, Lhasa, 850002 China; 3 Wuhan Igenebook Biological Technology Co., LTD, Wuhan, 430000 China

**Keywords:** Hulless barley, Genetic diversity, Population structure, Core germplasm, Association mapping

## Abstract

**Background:**

Clarifying genetic diversity in a large germplasm resource plays important roles in experimental designs that provides flexible utility in fundamental research and breeding in crops. However, the work is limited due to small collections of barley that are insufficient representatives.

**Results:**

In the present study, we collected 562 hulless barley (*Hordeum vulgare* L.) accessions with worldwide geographic origins and evaluated their genetic variability and relatedness based on 93 simple sequence repeat (SSR) markers. In an integrated analysis of the population structure, analysis of molecular variance (AMOVA) and pairwise *F*_ST_, the 562 barley accessions exhibited a strong stratification that allowed for them to be divided into two major subpopulations (p1 and p2) and an admixture subpopulation, with 93, 408 and 61 accessions, respectively. In a neutral test, considerable proportions of SSR alleles expressed the strong non-neutrality in specific subpopulations (44 and 37), which are probably responsible for population differentiation. To reduce the diversity redundancy in large barley collections, we delicately selected a core set of 200 barley accessions as a tradeoff between diversity and representativeness in an easily handled population. In comparing the 562 barley accessions, the core barley set accounted for 96.2% of allelic diversity and 93% to 95% of phenotypic variability, whereas it exhibited a significant enhancement in minor allelic frequencies, which probably benefit association mapping in the barley core set.

**Conclusions:**

The results provided additional insight into the genetic structure in a large barley germplasm resource, from which an easily manageable barley core set was identified, demonstrating the great potential for discovering key QTLs and ultimately facilitating barley breeding progress.

**Electronic supplementary material:**

The online version of this article (10.1186/s12863-017-0563-x) contains supplementary material, which is available to authorized users.

## Background

Hulless barley (*Hordeum vulgare* L.) with naked caryopsis is mainly distributed in Tibet and nearby areas. This was believed to be one of the important centers of origin of cultivated barley [1]. The hulless barley has become one of the most important staple crops in the Tibet region for the last 3500―4000 years because it is capable of adapting to the extreme climates and high altitudes. To date, hulless barley has predominantly occupied over 70% of the crop lands in Tibet [2], while it is also rarely distributed in Europe, the USA and Australia, as an important resource to feed animals. In recent years, hulless barley has proved its nutritional and economic value due to its high β-glucan content, which inhibited cholesterol synthesis, and the absence of grain husks probably reduced the processing cost of the barley food industry.

In breeding programs, a priori knowledge of genetic diversity and pairwise relatedness provides beneficial clues for efficiently utilizing a large collection of genetic resources. In the Himalayan region, particularly in Tibet, hulless barley showed a considerable contribution to the diversity of the barley germplasm worldwide. To date, there have been many studies reporting the genetic and phenotypic variability harbored in Himalayan barley accessions. In a survey of Nepalese naked barley germplasm, vastly morphologic [3] and disease-resistant [4] variations were found between and within the landrace populations. Recently, Pandey et al. [5] revealed a complex population structure of 107 hulless barley landraces, possibly due to the geographic differentiation among the barley populations. However, the observations in these studies are limited due to the small collections of barley and their insufficient representativeness.

The draft genomic sequence of the Tibetan hulless landrace “*Lasa Goumang*” offers opportunities to facilitate genetic improvement by the identification of gene or quantitative trait loci (QTL) of important traits [2]. It is therefore urgently important to build relevant populations for genetic mapping, i.e., linkage mapping and association mapping [6]. Comparatively, natural population-based genome-wide association studies (GWAS) are more powerful and cost-efficient than linkage mapping based on bi-parental crossing populations because the former usually accumulated more recombinant events and showed variation in their genomics and phenomics [7]. Importantly, the natural population confers a straightforward link between identification and breeding applications of key genes responsible for agronomic traits [8]. The trade-off between the experimental cost and mapping power is an advantage of the core germplasm set that harbors the majority (e.g., >90%) of the genetic variability of the total resource by a significantly reduced resource size, which would be an optimal choice in crop genetics. A number of GWAS on a manageable number of accessions (300―500) have been reported to dissect the genetic basis and even isolate single genes underlying key traits in rice [9, 10], maize [11], oilseed rape [12] and peanut [13]. In regards to breeding, we aim to identify the core germplasm that is capable of reducing the laboratory burden in encountering genetically redundant materials, which would increase breeding gain with limited cost.

In this study, we assembled a large-scale germplasm collection of 562 hulless barley accessions worldwide, but mainly in the Tibet region, which were genotyped using 93 simple sequence repeats (SSRs) dispersed across the whole genome. The objectives of this research are to (1) evaluate the genetic variability of the large hulless barley germplasm resource, (2) investigate the population structure and differentiation, and (3) identify and evaluate the utility of an optimal core set in hulless barley.

## Methods

### A collection of worldwide hulless barley accessions

**Fig. 1:**
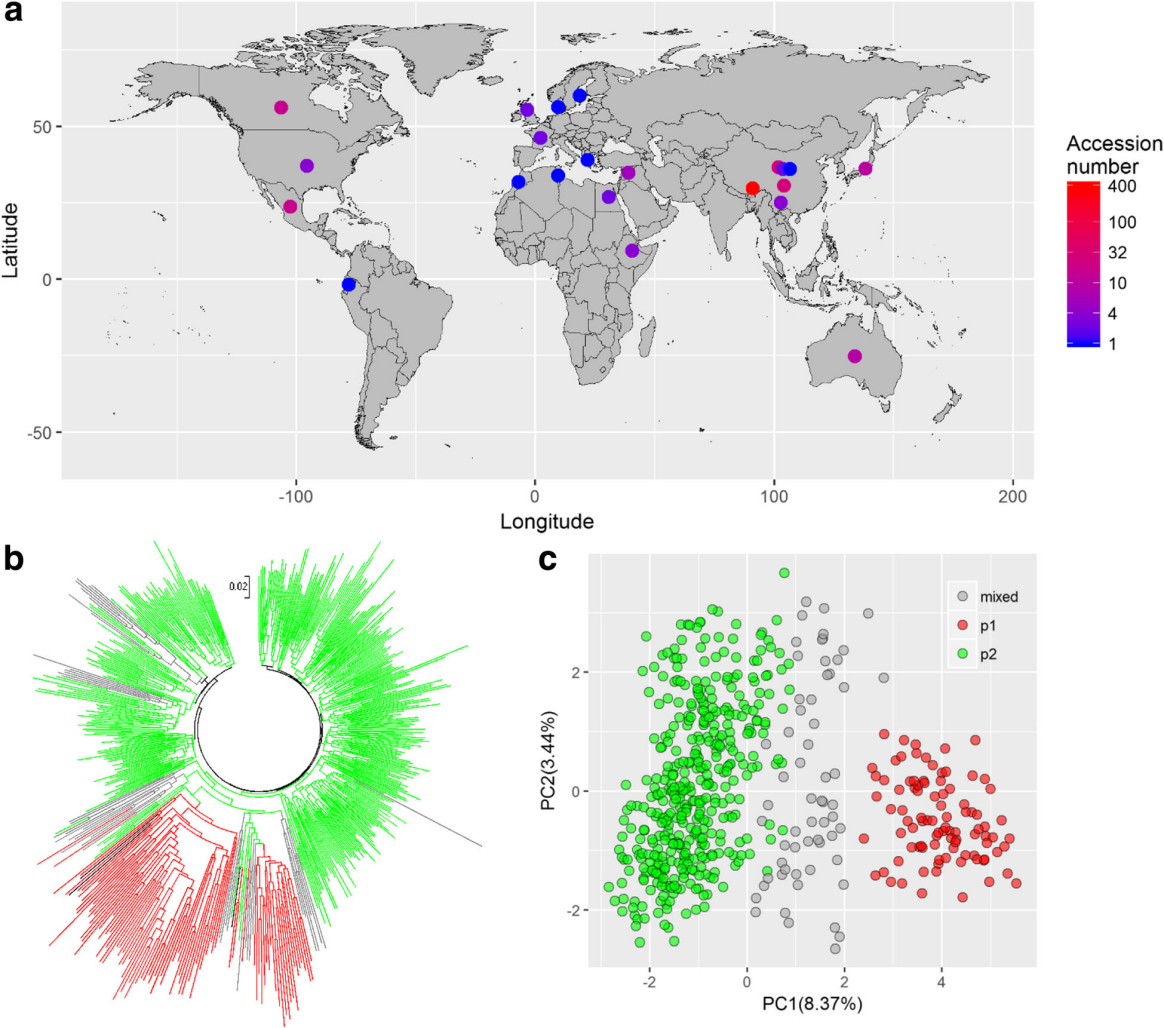
Geographic coordinates, phylogenic and principal component analyses of the 562 hulless barley accessions. **a** Colored dots refer to the sampling regions of the barley accessions, while the color gradient corresponds to the number of accessions sampled in each region. **b** All 562 barley accessions were clustered into two apparent clades, colored red and blue, based on the structural analysis. **c** The first two principal components (PC) explained over 10% of the molecular variance and discriminated the whole population into three groups, which is largely consistent with the subpopulations inferred by structural analysis

In this study, a set of 562 naked barley accessions were collected worldwide (Fig. 1a) the majority of which were derived from the Himalayan region, typically in China. This included 426 accessions from Tibet, 29 from Qinghai, and 27 from Sichuan. The remaining accessions were diversely dispersed across different continents (Fig. 1a). All barley accessions involved 98 cultivated varieties, 414 landraces and 50 wild resources. The materials are provided by Chinese Academy of Agricultural Science (CAAS) and accessible in the National Infrastructure of Plant Germplasm Resource in China (http://www.cgris.net/pt/). Detailed information about the 562 hulless barley accessions is listed in Additional file 1.

### SSR genotyping

For each accession, the genomic DNA was extracted from the young leaf tissues derived from a single plant following the modified cetyltrimethylammonium bromide (CTAB) protocol [14]. The quality of the DNA was evaluated on a 1% agarose gel through comparison with the uncut lambda DNA.

A set of 100 SSR primers were used in this study. Of these, 23 primers were retrieved from the public Graingenes 2.0 database (http://wheat.pw.usda.gov/GG2/) and 77 primers were newly developed by our laboratory based on the hulless barley reference genome [2]. First, 10 randomly selected barley accessions were used to test the polymorphism and amplification efficiency in all 100 SSR primers. Subsequently, all 562 hulless barley accessions were genotyped using 93 high-quality SSR primers, including 22 SSR primers from the public wheat database and 71 newly developed SSRs with the prefix “QK”. The information on the 71 new SSR primers is listed in Additional file 2. The polymerase chain reaction (PCR) analysis was performed according to the previous protocol [15], except that the SSR primers were labelled with fluorescent dyes. The SSR polymorphism was assayed by capillary electrophoresis (ABI 3730 Genetic Analyzer Applied Biosystems). The SSR primer alleles were quantified based on electrophoretic data using the GeneMarker V2.1 software. To avoid genotyping artifacts influenced by the SSR paralogs [16], we recoded the SSR alleles in binary data (1 or 0) to indicate the presence or absence of a certain allele in the whole population. To facilitate data analysis, the binary data per allele was treated as a dominant locus. The statistical value of the SSR primers was defined as an averaged value across all the alleles in a specific SSR.

### Genetic diversity

For each SSR primer, we calculated the allelic count, gene diversity (or expected heterozygosity) and polymorphism content (PIC) using PowerMarker version 3.51 [17]. The Shannon index per SSR was calculated using PopGene version 1.31 software [18]. Given the population size affect, the allelic count in a certain population and the allelic richness per SSR were calculated by dividing the total allelic count per SSR by the population size. The differences in the gene diversity, PIC, and allelic richness between the inferred subpopulations was evaluated based on the Wilcoxon’s paired test implemented in SAS 8.02 [19].

### Population structure and differentiation analyses

The STRUCTURE v2.2 software [20] based on the Bayesian model was used to explore the population structure of 562 barley accessions based on 93 SSRs. We simulated the number of subpopulations (*k*) from one to ten, with five replications. For each replication, the posterior probability, LnP(D), of the model was estimated based on 10,000 MCMC (Markov Chain Monte Carlo) iterations before using a burn-in length of 10,000 times with the admixture and related frequency model. The optimal *k* value was determined by the LnP(D) value with an ad hoc statistic delta *k* based on the rate of change in the LnP(D) between successive *k* [21]. Each hulless barley accession was assigned to corresponding subpopulations based on its maximum membership probability. Otherwise, the accession was classified into a “mixed” subpopulation if all the membership probabilities were below 0.65 [22]. The inferred subpopulations based on the STRUCTURE analysis were deployed in the following analysis.

To better illustrate the pairwise relationship across 562 hulless barley accessions, we performed principal component analysis (PCA), which was implemented in SAS 8.02 [19], and a neighbor-joining (N-J) tree based on the Nei’s distance using MEGA 4.0 [23]. To evaluate the dependency between the population structure and several clustering factors, such as geographic origin, eco-type, row-type and evolutionary type, the contingency table test based on an R function “chisq.test” was used (*P* < 0.05). Based on the subpopulations inferred by structural analysis, we carried out analysis of molecular variance (AMOVA) to assess the population differentiation using Arlequin V3.1 software [24], with 1000 permutations and a molecular distance based on the sum of squared size differences. On the other hand, the pairwise differentiation between subpopulations was evaluated by pairwise *F*_ST_, a measurement of expected heterozygosity within subpopulations compared to the total population [25].

To detect the link of population structure with allelic selection, we performed the Ewens-Watterson’s neutrality test [26, 27] for each binary locus (i.e., the SSR allele) in each subpopulation using PopGene version 1.31 software [18]. This was used to test whether the observed allele frequency is significantly higher or lower than the expected frequency by simulation under neutrality expectations [26, 27].

### The assembly of hulless barley core set

We used a multi-purpose core selection method to assemble the diverse core subset from the large germplasm collections with genome-covered SSR genotypes, implemented in the R package ‘corehunter’ [28]. To perform a GWAS in the core set, the high power of identifying strong signals necessitates sufficient allele repeats that are linked with interested phenotypes in a given population [6]. In this study, we expected a barley core set of 200 accessions, as the equivalent size has shown sufficient power in identifying major QTLs in self-mating species [10]. We deployed two parameters and weighted them equally to evaluate the hulless barley collection iteratively: (1) average entry-to-nearest-entry distance (EN), the maximization of which increased the average distance between each selected barley and the closest other selected item in the core; (2) gene diversity, which typically reflects the frequency to a balanced extent because the allele count in a specific locus was constantly two in this study. The optimal core set would potentially exhibit a high allelic diversity and low redundancy between any individual pairs. To independently evaluate the reliability of the assembled hulless barley core set, we deployed four agriculturally important traits (plant height, ear length, grain length and thousand seed weight) to assess the ability to maintain the phenotypic variability compared to the original large collection. Finally, we selected several informative SSRs to establish the fingerprints of the hulless barley core set with 200 accessions.

## Results

### Population structure and genetic diversity in the 562 hulless barley accessions

The population structure of a worldwide collection of 562 hulless barley accessions was investigated using 93 SSR markers. The maximal posterior probability (LnP(D)) of the Bayesian model was estimated at the possible subpopulations (*k*) from 1 to 10. We found that the most apparent change of the LnP(D) appeared when *k* increased from 1 to 2 (Additional file 3a). In addition, a sharp peak in delta *k* appeared at *k* = 2 (Additional file 3b). Accordingly, the 562 barley accessions were classified into two major subpopulations, designated p1 and p2, as well as a mixed subpopulation (Additional file 3c). The p1 subpopulation contained 93 accessions, of which, 85 accessions were from China (78 from Tibet), 2 were from Australia, 4 were from Canada and 2 were from Mexico. In p1, there were 84 barley landraces, 7 barley cultivars and 2 wild barley resources. The p2 subpopulation contained 408 barley accessions, of which, 344 accessions were from China (296 from Tibet), 14 were from Mexico, 12 were from Canada, 10 were from Japan, 7 were from Australia and 1―5 were from other East Asia countries. In p2, there were 289 barley landraces, including 76 cultivars and 43 wild accessions. The mixed subpopulation contained 61 barley accessions, almost covering all the geographic regions sampled in the present study. The information on the geographic origins, types and inferred subpopulations is listed in detail in Additional file 1. We further employed N-J phylogenetic analysis and principal component analysis (PCA) to determine the genetic relationship across the 562 barley accessions. All 562 barley accessions were clustered into two apparent clades corresponding to the subpopulation determined by structural analysis (Fig. 1b). This pattern was additionally verified by the PCA plot, in which the top two principal components accounted for over 10% of the molecular variance (Fig. 1c). The contingency table test revealed that the inferred population structure was significantly correlated to the evolutionary barley types (*P* = 0.028). Specifically, the p2 subpopulation was enriched in wild barley relative to the p1 subpopulation (Additional file 1). Therefore, the two major structural populations were used in following analyses.

The genetic diversity in the total barley collection and inferred subpopulations were measured by the allele number, allelic richness, gene diversity, polymorphism information content (PIC) and Shannon index. A summary of the diversity analysis per SSR is listed in Additional file 4. In the total collection, the 93 SSR markers detected 516 alleles, with 5.55 alleles per SSR. The gene diversity, PIC and Shannon index were 0.189 (0.009–0.499), 0.157 (0.009–0.374) and 0.305 (0.029–0.692), respectively (Table 1 and Additional file 5). The p1 and p2 subpopulations contained 456 and 467 alleles, with 4.9 and 5.0 alleles per SSR, respectively. The number of alleles in p2 was slightly higher than that in p1, although the sample size of p2 was over fourfold that of p1. On the other hand, p1 had a similar number of alleles per SSR to p2 but significantly more allelic richness than p2 (*P* < 0.01). The level of gene diversity, PIC and Shannon index consistently were consistently higher in p1 than in p2, but the extent varied slightly (Table 1 and Additional file 5). The mixed subpopulation had almost one-seventh the sample size in p2 but harbored a comparable number of alleles, which probably led to the highest level of allelic richness in the mixed subpopulation (Table 1).

**Table 1:** Diversity in barley structurally inferred subpopulations

	Total	p1	p2	Mixed
Sample size	562	93	408	61
Alleles	516	456	467	446
Alleles/SSR	5.55(2–16)	4.9(2–14)	5.02(2–15)	4.8(2–13)
Allelic richness^a^	0.918149466	4.903225806	1.144607843	7.31147541
Gene diversity	0.189(0.009–0.499)	0.206(0.007–0.489)	0.162(0–0.491)	0.211(0.011–0.496)
PIC	0.157(0.009–0.374)	0.169(0.007–0.369)	0.134(0–0.37)	0.173(0.011–0.373)
Shannon index	0.305(0.029–0.692)	0.326(0.02–0.682)	0.26(0–0.684)	0.332(0.028–0.689)

^a^Allelic richness indicated the average allele count per accession in the whole population and subpopulations, which was calculated as the total allele count within a population divided by population size

### Genetic differentiation and neutrality between inferred subpopulations

We performed AMOVA and pairwise *F*_ST_ to investigate the population differentiation between the inferred subpopulations. AMOVA results illustrated that 16.07% (*P* < 0.001) of the total molecular variation in the 562 hulless barley accessions was attributed to genetic differentiation between inferred subpopulations (Table 2). Pairwise *F*_ST_ between the p1 and p2 subpopulations was 0.223 (*P* < 0.001), but the values between the mixed and p1 and the mixed and p2 were 0.079 and 0.075 (*P* < 0.001), respectively (Additional file 6). This suggested that p1 is significantly divergent from p2, but they were relatively genetically similar to the mixed subpopulation. A similar pattern of genetic differentiation among the inferred subpopulations was confirmed using the results of the pairwise Nei’s minimum distance (Additional file 6). The correlation coefficient between the *F*_ST_ and Nei’s distance was 0.99 (*P* < 0.01).

**Table 2:** Analysis of molecular variance (AMOVA) among the inferred barley subpopulations based on STRUCTURE analysis

Var. Source	d.f.^b^	SS	Var. Comp.	Variance(%)^c^
Among subpopulations^a^	2	4393.611	8.82205	16.07**
Within subpopulations	1121	51,653.286	46.07786	83.93**
Total	1123	56,046.897	54.89991	

**P < 0.001, for 1000 permutations

^a^Subpopulations were defined by structure analysis when *k* = 2, including p1, p2

^b^Stands for the degree of freedom

^c^The percentage of molecular variance attributed to the variance among and within the subpopulations

**Fig. 2:**
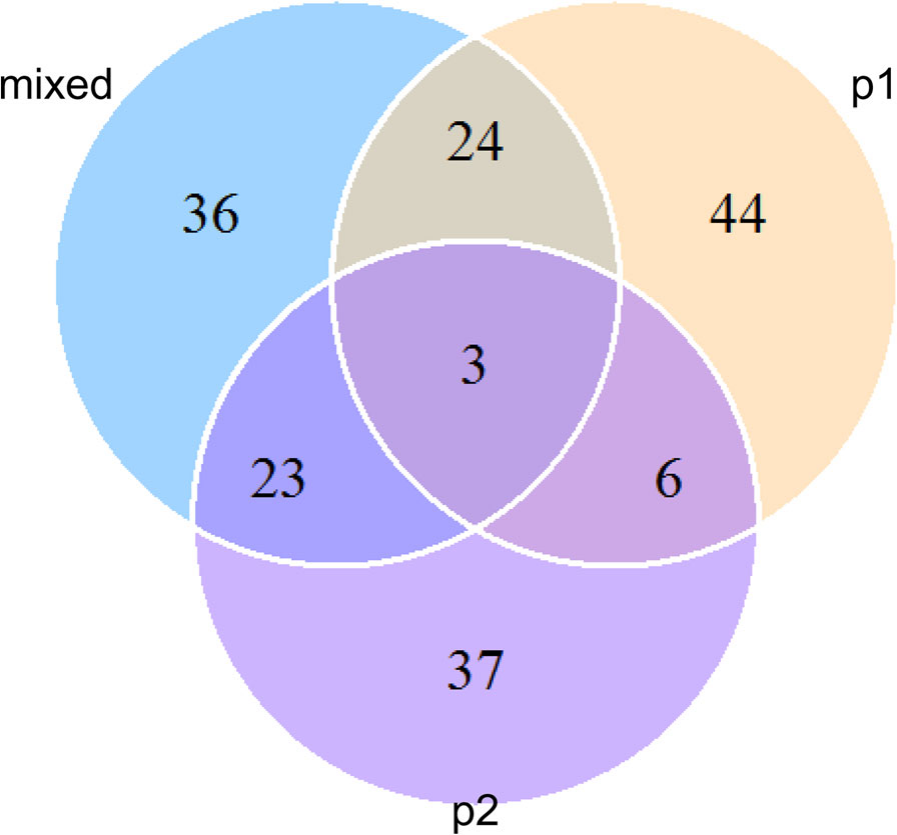
Genetic differentiation between the subpopulations was revealed by the neutrality analysis. The number labeled within the different sections of the Venn-plot represent the number of microsatellite alleles that showed significantly non-neutral frequency in specific subpopulations or shared between subpopulations

To further evaluate the influence of population structure, we deployed the Ewens-Watterson’s neutrality test to determine whether there are specific SSR alleles favored in the subpopulations. In summary, we found that there were 173 SSR alleles that showed significantly higher or lower frequencies than the expected value based on the neutral model (*P* < 0.01) (Fig. 2). The non-neutrality of these SSR alleles may be attributed to directional selections linked to population differentiation. Of all the non-neutral SSR alleles, there were 77 (44.5%), 69 (39.9%) and 86 (49.7%) alleles that were significantly discovered within p1, p2 and mixed subpopulations, respectively. We found that 9 non-neutral alleles were shared between p1 and p2, while 27 and 26 non-neutral alleles were shared between p1 and mixed and between p2 and mixed, respectively. Moreover, a considerable proportion of the non-neutral alleles that were specific to the subpopulations were identified, with nearly 25.4% and 21.4% of the non-neutral alleles being specific to p1 and p2, respectively (Fig. 2).

### The assembly and characteristics of barley core resource

Considering the need for GWAS in barley, in this study, we assembled a core set of 200 hulless barley accessions (~1/3 of original population size) to sustain sufficient mapping power but largely reduce genotyping and phenotyping cost. The identifiers of the 200 hulless barley core accessions are indicated in Additional file 1. We found that the core accessions were evenly distributed across the raw collection of 562 accessions that were consistently revealed by the NJ phylogenic and PCA analyses (Additional file 7). Specifically, in the hulless barley core set, 34 accessions were selected from the p1 subpopulation, 148 from the p2 subpopulation and 18 from the mixed subpopulation, accounting for 29.5%–36.6% of the accessions per subpopulation.

**Fig. 3:**
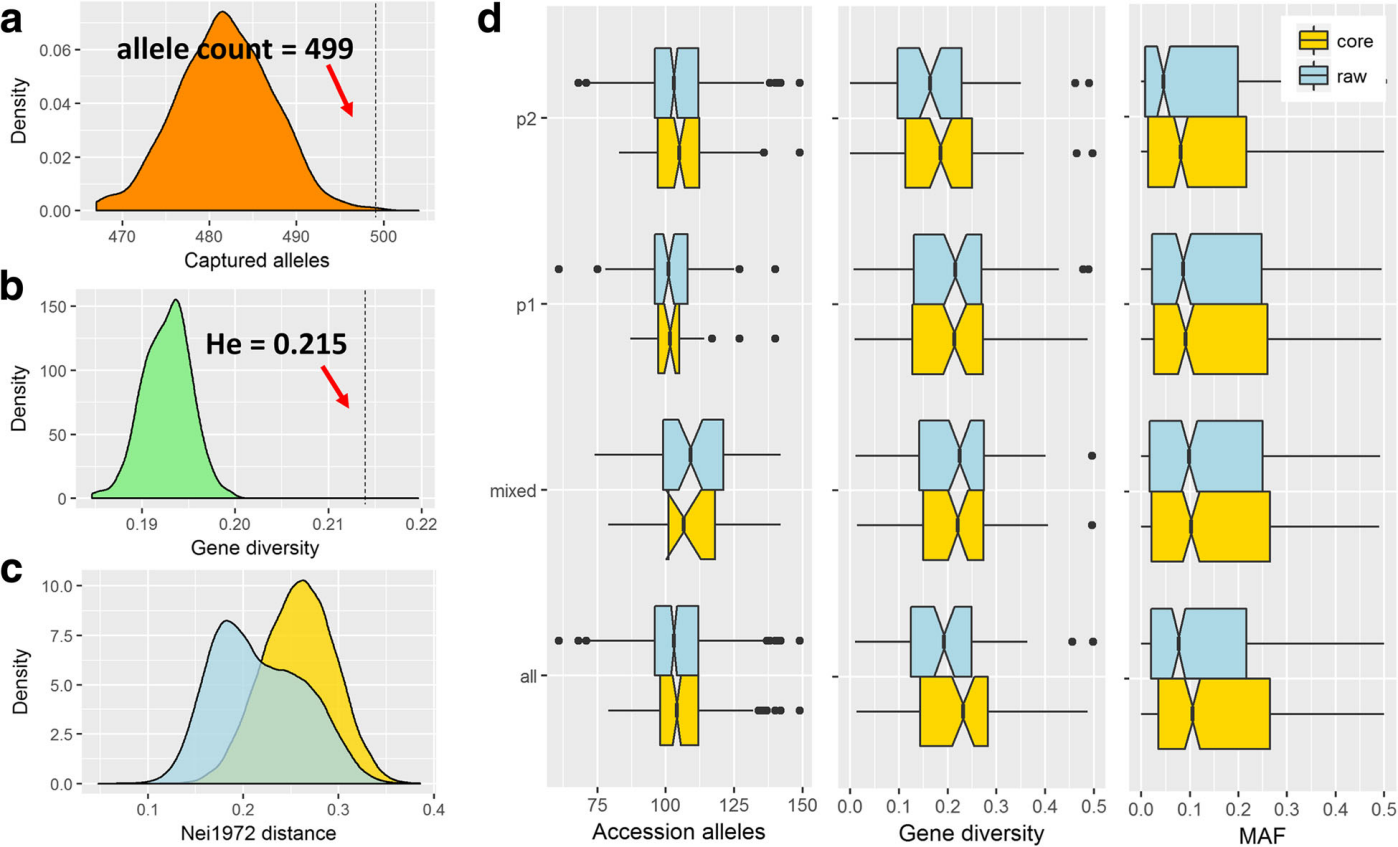
Diversity analysis of the assembled barley core set. The barley core set of 200 accessions was mathematically selected from all 562 accessions using the R package ‘corehunter’. **a** The selected barley core set captured 499 microsatellite alleles accounting for 96.7% of the alleles harbored in the 562 accessions, which is significantly more than the 200 random accessions. **b** Gene diversity of the core set averaged across the SSR primers. It is significantly higher than the raw 562 accessions and 200 random accessions, which is probably due to the high allele maintenance and balanced allelic frequency in the core set. **c** Comparison of the Nei1972 distance for accession pairs between the barley core set and raw set; **d** The alleles captured by accession per se (*left panel*), gene diversity per SSR (*middle panel*) and minor allele frequency (MAF) of the alleles (*right panel*) for each subpopulation and whole population in the core (200 accessions) and raw (562 accessions) sets

To evaluate the reliability of the hulless barley core set, we compared the diversity between the core set and random sets assembled by chance with 1000 repeats. We found that the core set significantly captured more alleles (499 vs. 482; *P* = 7.8E-4; Fig. 3a) with a higher gene diversity (0.215 vs. 0.195; *P* = 9.3E-18; Fig. 3b), indicating that the barley core set is a reasonable outcome that is significantly better than random events. Interestingly, the assembled core selection had moved the average pairwise distance between the accessions from 0.17 to 0.26 (Fig. 3c), which potentially eliminated duplications and highly similar accessions, resulting in a highly representative assembled barley core set. In summary, the barley core set contained a total of 499 alleles, accounting for 96.7% of the existing alleles in the original 562 accessions. Typically, the allelic count per accession was comparable between the barley core and raw set in either subpopulation or the whole population (*P* > 0.05; Fig. 3d). For the gene diversity, the overall diversity value across all the SSRs was 0.221 in the core set, which is significantly beyond the value of 0.189 that was observed in the raw set (*P* = 0.028). The gene diversity was not significantly different between the barley core and raw set in any subpopulation, but p1 had a marginally higher diversity in the barley core set than in the raw set (Fig. 3d). The allelic frequency exhibited a similar trend with gene diversity, probably explaining that the overall diversity of the barley core set increased compared to the raw set. The summary of the diversity per SSR in the hulless barley core set is listed in Additional file 4.

To evaluate the maintenance of the phenotypic variability in the core set, we compared the hulless barley core and raw set in the four agronomic traits. We found that the ear length, grain length and the thousand seed weight showed a slightly higher coefficient of variation (CV) but plant height had a slightly lower CV in this barley core set than the raw set (Table 3). In addition, the Kolmogorov-Smirnov test revealed that all four traits followed a non-significantly different distribution (i.e., statistically approximately identical) between the core and raw sets (Additional file 8). It demonstrated that the barley core selection based on genetic data largely maintained phenotypic variability.

**Table 3:** Comparison of the coefficient of variation for the phenotypic value between the core barley set and the raw 562 barley accessions in this study

		Coefficient of variation			
	n	Ear length	Grain length	Thousand seed weight	Plant height
Core set	200	0.2434	0.1391	0.1485	0.1814
Raw set	562	0.2424	0.1314	0.1471	0.1844

Regarding the aims in practice, we used ten informative SSR primers that had top10 diversities (see Additional file 4 in details) to construct the fingerprint of each accession in the barley core set (Additional file 9). The fingerprint provided an efficient way to discriminate identities of the barley core set in the breeding program and seed market.

## Discussion

### Genetic diversity and population structure in the large hulless barley resource

Understanding of the genetic diversity and population structure provides an opportunity to evaluate the applicable potential of a new germplasm resource in the breeding aims and fundamental studies. In this study, we employed 93 SSR primers to evaluate the genetic diversity in a large hulless barley collection of 562 accessions. On average, the SSR amplified 5.55 alleles per SSR, with a range from 2 to 16 alleles per SSR, which was comparable to the value (5.54 in average) of the 44 SSRs in the 107 hulless barley landraces collected from the highlands of Nepal [5]. However, the PIC value was comparatively lower in this study, which may be partly explained by three reasons: (1) the difference in the SSR primers used in two studies; (2) the difference in the PIC calculation algorithm, including the decomposition of the multi-allelic SSRs into the bi-allelic loci, which would unavoidably result in a loss of information that may be proportional to the allele count; (3) the difference in the germplasm resource, including more barley landraces and wild type that significantly enriched the gene library, while the exotic germplasm potentially introduced private or rare alleles (more discussed hereafter).

The Bayesian-model-based structure analysis is widely used a method for the inference of hidden population structure in human and plant species [29, 30]. In this study, two major subpopulations were identified in the collection of 562 hulless barley accessions, the structural pattern of which was verified by the NJ phylogenic and PCA analyses (Fig. 1). Interestingly, we found that the two subpopulations were significantly correlated with the evolutionary types of the barley accessions (*P* = 0.028). The p2 population contained 10.5% of the wild barley accessions, nearly 5 fold more than p1. This difference may be reflected by the fact that the Himalayan vicinity is one of the centers for barley origin in which the majority of accession regions in p2 were collected. However, the two major subpopulations were not significantly correlated to the geographical origins, row-types and eco-types (Additional file 1). Given that the delta *k* additionally peaked at a *k* of 5 and 7, respectively (Additional file 3b), we therefore re-clustered the whole population into 5 and 7 subpopulations. It was probably a refinement of two major subpopulations that was identified in *k* = 2 (Additional file 10), which enabled these subpopulations to be significantly correlated to the geographic origins (*P* = 9.38E-7 and 1.78E-11) and row-types (*P* = 0.032 and 0.017) at *k* = 5 and 7, respectively (Additional file 11). In the following analysis, we focus on the major two subpopulations that explained a large variance in the population differentiation. We found that the alleles per SSR for the total population and two inferred subpopulations were comparable, but p2 had almost one-fifth the allelic richness, while it had over fourfold the sample size compared to p1 (Table 1). This suggested that, in the p2 subpopulation, the large amounts of exotic barley landraces and wild types may prevalently capture private alleles (the allele that existed specifically only in one accession) or rare alleles. It is reliable because all the diversity measurements (gene diversity, PIC and Shannon index) were consistently significantly lower in p2 compared to p1 (Table 1 and Additional file 4) and the median minor allele frequency (MAF) values were significantly lower in p2 than p1 (Fig. 3d).

### Perspectives of the assembled hulless barley core set

In plant breeding, the core collection plays important roles in parent selection for cross breeding and seed conservation. The core collection aims to maximize the genetic diversity of a large germplasm resource in a manageable sized subset, the operation of which will largely reduce redundant labors in the limited breeding cycles. In this study, we exhaustively tested all the possibilities and selected the optimal one (core set), which simultaneously reached a maximum allele amount and genetic diversity. The barley core set captured a total of 96% of the SSR alleles that existed in the raw population but significantly elevated the gene diversity level. Additionally, the barley core set almost mimicked the phenotypic distribution of the four agronomic traits and retained the phenotypic variability of the original 562 hulless barley accessions. These findings demonstrate that the hulless barley core set represented sufficient variation in the genetic and phenomic layers.

GWAS has been increasing in popularity for unraveling the genetic basis of simple and complex traits in plants [31, 32]. Presently, the reference genome of the Tibetan hulless landrace “*Lasa Goumang*” has been released [2]. It opens an era of GWAS-based gene identification and genomic selection (GS) based on genetic improvement in barley breeding, as tremendous molecular markers would be easily retrieved by resequencing and bioinformatic analysis [33, 34]. The establishment of the multi-ends research population is significantly important in the barley research community. The use of a carefully selected core set of diverse accessions is a routine approach to facilitate GWAS [33], which is probably due to the trade-off between the mapping power and labor- and cost-requirement. However, many researchers argued that the naturally-existing population always harbors numerous rare variations, which probably missed the heritability as given by the low frequency variation, which is difficult to detect statistically by GWAS [35, 36]. Interestingly, to this point, the barley core set provided an optimal mapping population for GWAS, because it significantly balanced the allelic frequencies, elevating the median MAF from 0.077 to 0.11 (all) and from 0.045 to 0.081 (p2) (Fig. 3d), which potentially boosted the mapping power imbedded in this barley core set. In summary, the multi-end use of the core set provided a feasible way to bridge traditional and molecular breeding in hulless barley.

## Conclusions

In this manuscript, we collected 562 hulless barley accessions with worldwide geographic origins (the largest collection to date) and evaluated their genetic variability and relatedness based on 93 SSR markers. The joint analyses of PCA, AMOVA, and pairwise-F_ST_ and the neutrality test consistently verified the reliability of the population structure. Our results provided new insights into the genetic structure in a large hulless barley germplasm resource. Furthermore, we identified an optimal core set of 200 hulless barley accessions with a high maintenance of the genomic and phenomic diversity. The barley core set enabled the reform of the allelic frequency, which potentially boosted power in discovering key QTLs and ultimately facilitating progress in barley breeding.

## Additional files

Additional file 1: Table S1. Accession information of 562 hulless barley accessions in the present study. (XLSX 57 kb)

Additional file 2: Table S2. Information of the 73 SSR primers based on the hulless barley reference genome. (XLSX 14 kb)

Additional file 3: Figure S1. Structural analysis of the 562 barley accessions based on the Bayesian model. a) the LnP(D) plot against the k series; b) the delta k plot against the k series; c) bar-plot of 562 barley accessions representing the membership probability of the inferred subpopulations. At the probability cutoff of 0.65, the total population was divided into two subpopulations, p1 (①) and p2 (②), appending the mixed subpopulation (③). (TIFF 1187 kb)

Additional file 4: Figure S2. Information of the genetic diversity for the SSR markers used in this study. (XLSX 44 kb)

Additional file 5: Table S3. Boxplots for the comparison of the genetic diversity across the inferred subpopulations. (TIFF 4562 kb)

Additional file 6: Table S4. Genetic differentiation coefficient (F_ST_) and Nei1972 distance between the pairwise structural inferred subpopulations. (XLSX 8 kb)

Additional file 7: Figure S3. Genetic distribution of 200 barley core accessions based on the phylogenic and principal component analysis. a) in the phylogenic tree, the clades were colored by structural results, i.e., red and green colors indicate the accessions from the p1 and p2 subpopulations, respectively, while gray to the accessions from mixed population; the clades labeled with black dots refer to the 200 barley core accessions; b) in the PCA plot, the red points refer to the 200 barley core accession. (TIFF 4117 kb)

Additional file 8: Figure S4. Phenotypic distribution of the four agriculturally important traits in the core and raw barley sets. (TIFF 88 kb)

Additional file 9: Table S5. Information on the fingerprint identifiers for the 200 barley core accessions. (XLSX 13 kb)

Additional file 10: Figure S5. Refinement of the population structure. A1-A2, indicates 2 subpopulations at K = 2; B1-B5, indicates 5 subpopulations at K = 5; C1-C7, indicates 7 subpopulations at K = 7. (TIFF 3398 kb)

Additional file 11: Table S6. Significance of the correlations between the population structure and several clustering factors. The *p* values were calculated using the contingency table test. (XLSX 8 kb)

## Abbreviations

AMOVA: Analysis of molecular variance
CTAB: Cetyltrimethylammonium bromide
CV: Coefficient of variation
EN: Entry-to-nearest
GS: Genomic selection
GWAS: Genome-wide association study
MAF: Minor allele frequency
MCMC: Markov Chain Monte Carlo
NJ: Neighbor joining
PCA: Principal component analysis
PCR: Polymerase chain reaction
PIC: Polymorphism information content
QTL: Quantitative trait locus
SSR: Simple sequence repeats
