# Supplementary figures and images for: A microsatellite diversity analysis and the development of core-set germplasm in a large hulless barley (Hordeum vulgare L.) collection
Source: BMC Genet. 2017 Dec 6;18:102. doi: 10.1186/s12863-017-0563-x (PMC5717800; doi:10.1186/s12863-017-0563-x)

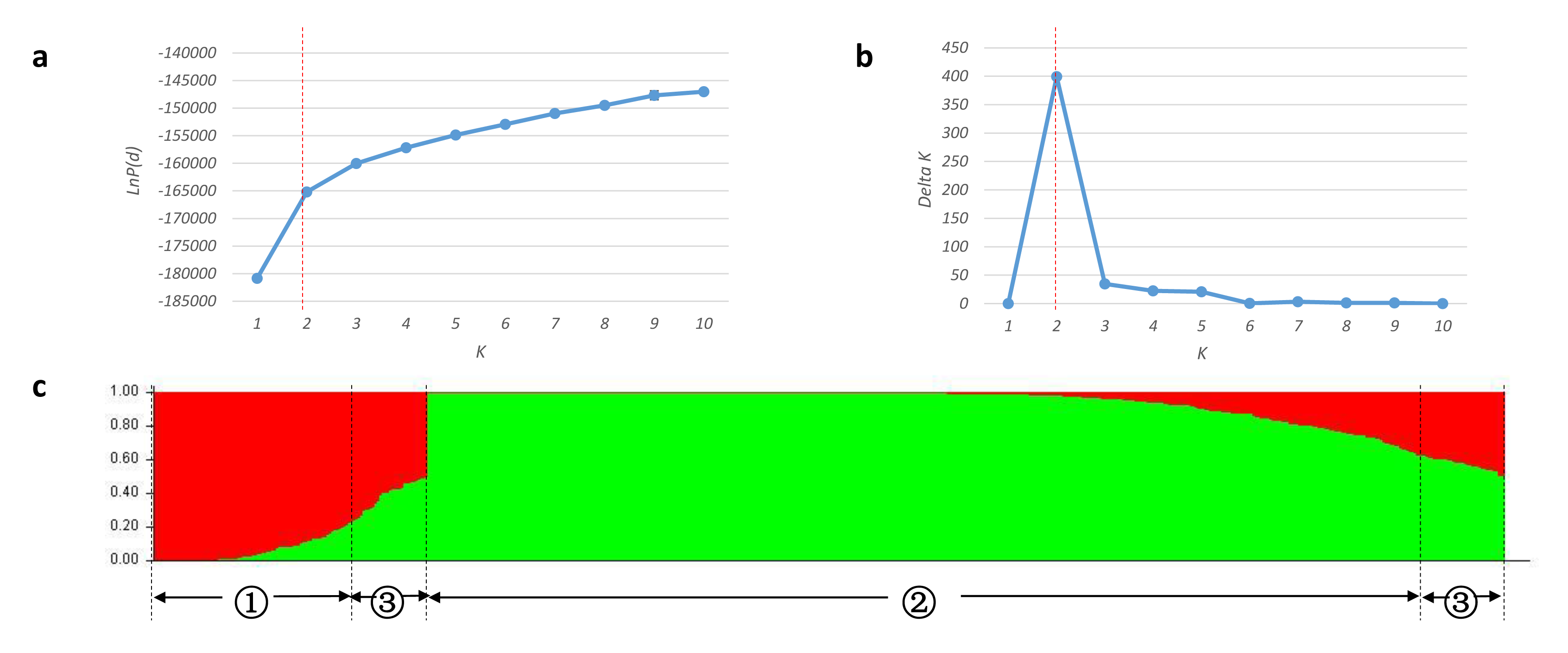

Supplement: Supplementary file 3 — Structural analysis of the 562 barley accessions based on the Bayesian model. a) the LnP(D) plot against the k series; b) the delta k plot against the k series; c) bar-plot of 562 barley accessions representing the membership probability of the inferred subpopulations. At the probability cutoff of 0.65, the total population was divided into two subpopulations, p1 (①) and p2 (②), appending the mixed subpopulation (③). (TIFF 1187 kb) [file 12863_2017_563_MOESM3_ESM.tif]

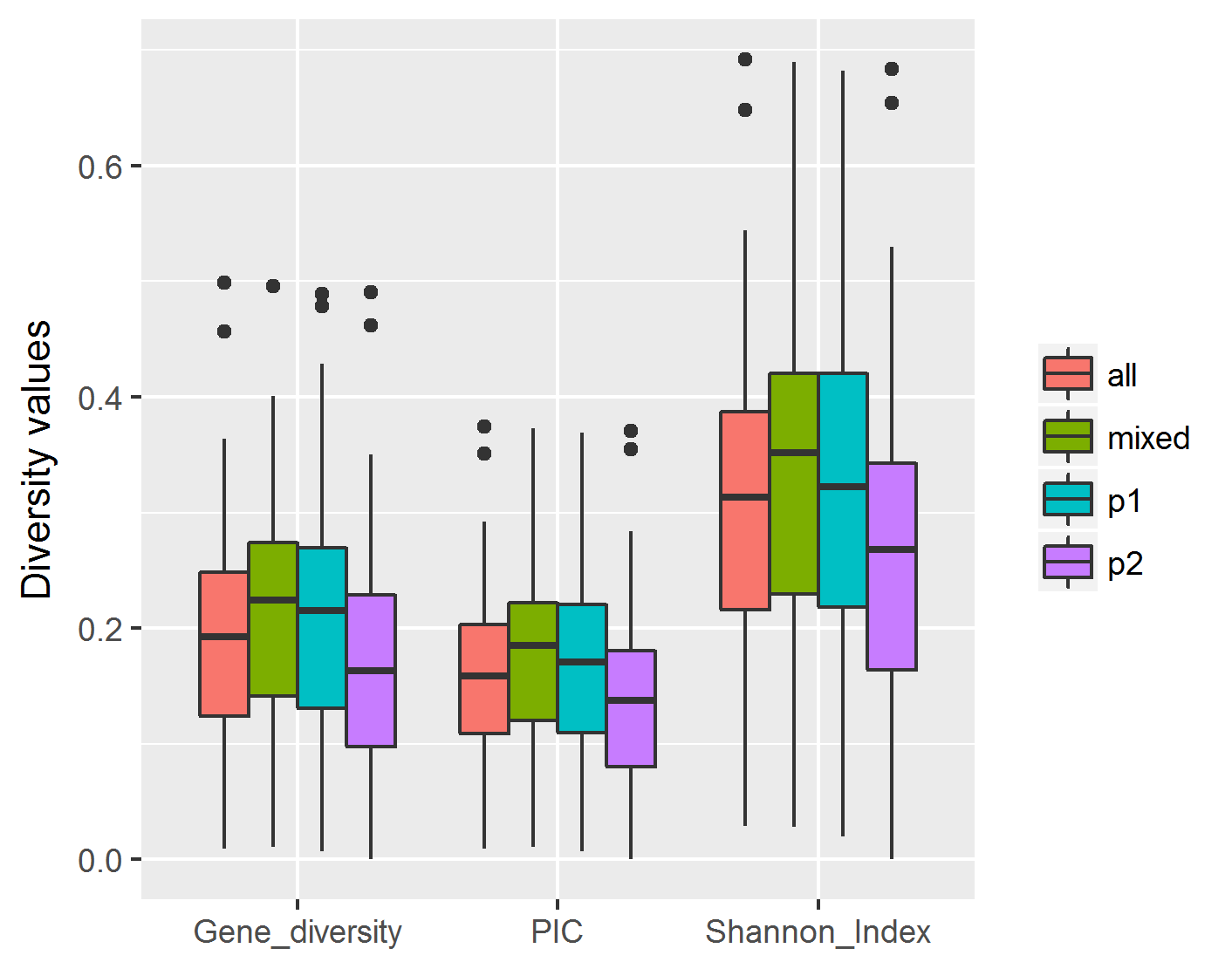

Supplement: Supplementary file 5 — Boxplots for the comparison of the genetic diversity across the inferred subpopulations. (TIFF 4562 kb) [file 12863_2017_563_MOESM5_ESM.tiff]

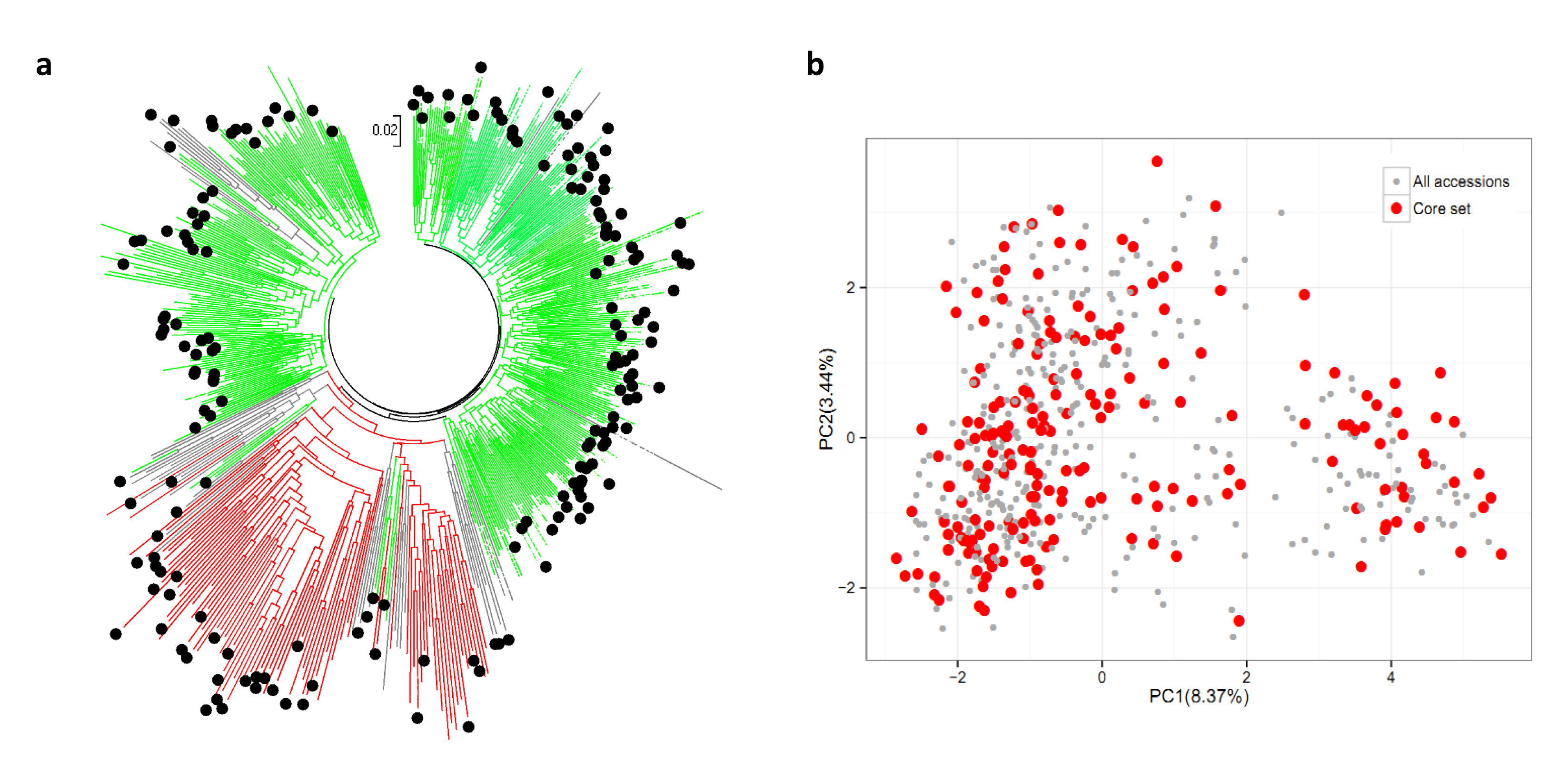

Supplement: Supplementary file 7 — Genetic distribution of 200 barley core accessions based on the phylogenic and principal component analysis. a) in the phylogenic tree, the clades were colored by structural results, i.e., red and green colors indicate the accessions from the p1 and p2 subpopulations, respectively, while gray to the accessions from mixed population; the clades labeled with black dots refer to the 200 barley core accessions; b) in the PCA plot, the red points refer to the 200 barley core accession. (TIFF 4117 kb) [file 12863_2017_563_MOESM7_ESM.tif]

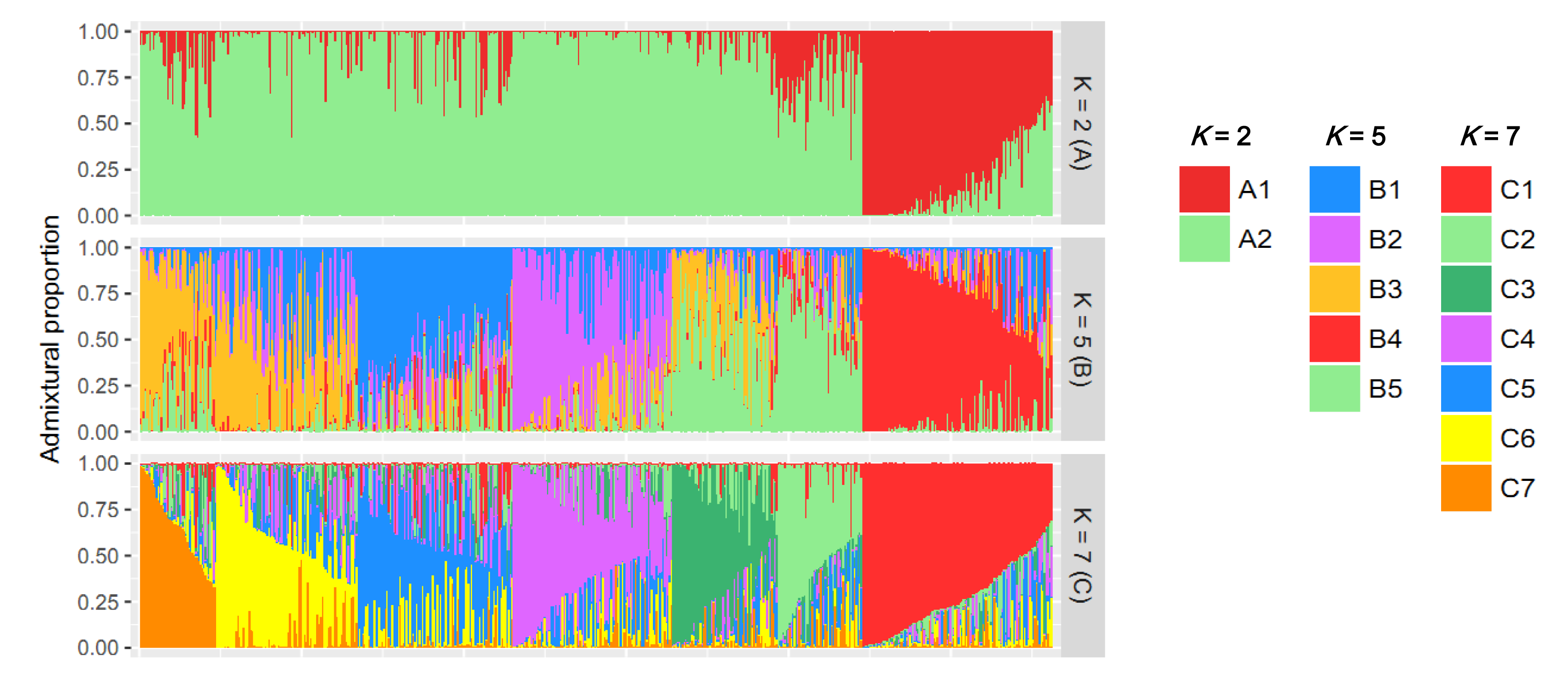

Supplement: Supplementary file 10 — Refinement of the population structure. A1-A2, indicates 2 subpopulations at K = 2; B1-B5, indicates 5 subpopulations at K = 5; C1-C7, indicates 7 subpopulations at K = 7. (TIFF 3398 kb) [file 12863_2017_563_MOESM10_ESM.tif]
